# Celebrating 20 years of collaboration to eliminate trachoma

**Published:** 2025-01-31

**Authors:** PJ Hooper, Michaela Kelly, Angelia Sanders

**Affiliations:** 1 Chair: International Coalition for Trachoma Control, Atlanta, USA.; 2 Vice Chair: International Coalition for Trachoma Control, Haywards Heath, United Kingdom.; 3 Immediate Past Chair: International Coalition for Trachoma Control, Atlanta, USA.

**This year marks the 20th anniversary of the International Coalition for Trachoma Control.**

**Figure:**
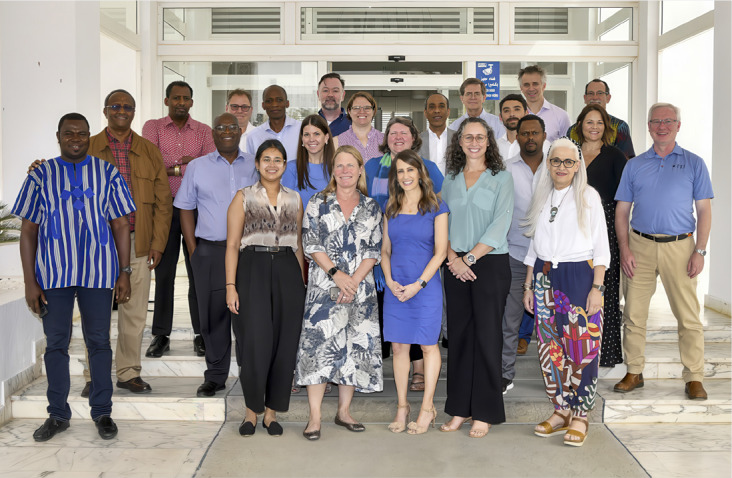
ICTC Membership Meeting, Hammamat, 2024. TUNISIA

© INTERNATIONAL COALITION FOR TRACHOMA CONTROL CC BY-NC-SA 4.0

The International Coalition for Trachoma Control (ICTC) is a global partnership that unites diverse stakeholders with a shared vision of eliminating trachoma as a public health problem by 2030.

Established in 2004, ICTC provides a platform for non-governmental organisations (NGOs), academia, donors, and private sector organisations to collaborate and support national trachoma programmes in advocating for and implementing the World Health Organization (WHO)-endorsed SAFE strategy (surgery, antibiotics, facial cleanliness, and environmental improvement).

In particular, ICTC members respond to priorities raised by countries through the WHO Alliance for the Global Elimination of Trachoma by 2020 by developing tools and resources to support SAFE strategy implementation. These resources include examples of supportive supervision and training for trachomatous trichiasis (TT) surgery, counselling guides for TT, and strategies for integrating trachoma interventions into routine eye health systems. ICTC also works with its member organisations to document programmatic lessons, including efforts to mainstream disability inclusion, promote gender equity, as well as developing strategies to expand eye health services to the most remote and marginalised populations, including refugees, internally displaced persons, indigenous groups, and nomadic communities.

Significant progress has been made since 2004 in eliminating trachoma as a public health problem. Notably, the number of people at risk of trachoma has decreased from 1.5 billion in 2002 to 103 million in 2024. This rapid progress has been made possible by significant investments to map the global burden of trachoma and the generous donation of azithromycin by Pfizer Inc., through the International Trachoma Initiative, to scale up mass drug administration in all trachoma-endemic areas.

These activities have leveraged further investment in SAFE strategy implementation, including two major partnership initiatives funded by the United Kingdom's Department for International Development and The Queen Elizabeth Diamond Jubilee Trust Trachoma Initiative, which provided ICTC with over $100 million to support national programmes to scale up all components of the WHO-endorsed SAFE strategy in select countries across Africa and the Pacific from 2014 to 2019. Through these partnerships, ICTC identified member organisations with relevant experience and established partnerships in each supported country to provide technical and financial assistance to the relevant ministry of health for a range of activities including programme design, training, community sensitisation, implementation, supervision, and monitoring and evaluation. These activities were delivered by each country's ministry of health through existing health structures to accelerate trachoma elimination while simultaneously strengthening health systems. In Africa, this investment supported over 213,000 TT surgeries, delivered 75.7 million antibiotic treatments in endemic areas, and implemented facial cleanliness and environmental improvement interventions across 153 districts. These partnership initiatives also catalysed newer investments, bringing together multiple ICTC members to deliver cross-sectoral trachoma programmes focused on impact, equity and sustainability.

In 2022, ICTC published its strategic plan 2022–2030, which lays out the coalition's strategic objectives to maximise its contribution towards the elimination of trachoma as a public health problem, and to achieve targets set in the WHO global road map for neglected tropical diseases 2021-2030 and the World Report on Vision. These include: mobilising advocacy efforts, increasing investment, coordinating technical assistance, and ensuring an effective coalition model. The document emphasises the importance of integrating interventions for trachoma into the broader national eye health services package to ensure the sustained impact of trachoma elimination through continuous availability and access to surgical services, screening, treatment and that continuity of care is built into national health systems.

Going forward, ICTC is committed to increasing and strengthening coordination with eye health systems to integrate trachoma interventions into routine health services where appropriate, and utilising trachoma systems to strengthen eye health systems where they are insufficient. As ICTC celebrates two decades of impactful collaboration, the coalition remains steadfast in its commitment to eliminating trachoma as a public health problem and improving global eye health, ensuring that the progress made continues to benefit communities worldwide for years to come.

